# Synonymous mutations in essential genes infrequently produce fitness effects in human cell lines

**DOI:** 10.1093/nar/gkag733

**Published:** 2026-08-05

**Authors:** Yiyun Rao, Ivan Sokirniy, Edward O’Brien, Justin Pritchard

**Affiliations:** Huck Institutes of the Life Sciences, Pennsylvania State University, University Park, PA 16802, United States; Huck Institutes of the Life Sciences, Pennsylvania State University, University Park, PA 16802, United States; Department of Chemistry, Pennsylvania State University, University Park, PA 16802, United States; Huck Institutes of the Life Sciences, Pennsylvania State University, University Park, PA 16802, United States; Department of Biomedical Engineering, Pennsylvania State University, University Park, PA 16802, United States

## Abstract

The assumption that synonymous mutations are fitness-neutral is central to many foundational results in the fields of genetics, genomics, evolutionary biology, and medicine. However, recent results suggest synonymous mutations have pervasive and strong fitness effects. These vigorously debated studies in non-human model systems have even suggested that the proportion of synonymous mutations and their fitness effect sizes are similar to non-synonymous mutations. To probe the fitness effect of synonymous mutations, we utilized recent advances in base editing to test 8558 potential synonymous mutations in 128 highly essential genes in human cell lines. Importantly, our library design excluded splice-proximal sites, ensuring a direct test of codon-level synonymous effects independent of splicing disruption. We find that synonymous mutations rarely have fitness effects on growth, occurring around 37.9-fold (95% CI: 22.16–81.48-fold) less frequently than missense mutations. In this experimental context, these findings demonstrate that synonymous mutations impact cellular fitness far less frequently than missense mutations. These results deviate from earlier reports of widespread synonymous fitness effects in yeast, yet they align with recent prime editing data observed in other human cell lines.

## Graphical Abstract

**Graphical Abstract:**
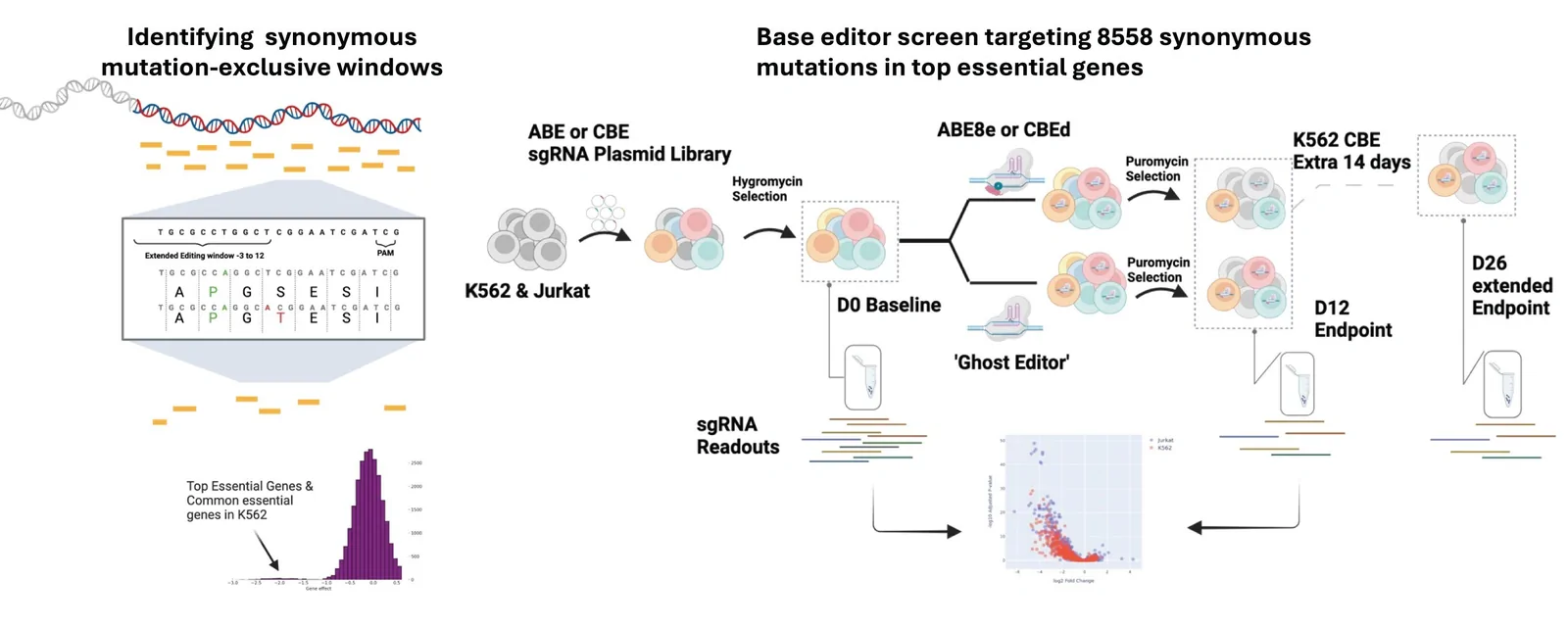

## Introduction

Synonymous mutations are nucleotide changes in codons that do not alter the encoded amino acid sequence. The assumption that synonymous mutations are largely invisible to natural selection is a cornerstone of modern protein-based phylogenetic models [1, 2]. It is embedded in landmark genomic annotation efforts [3, 4] and has been incorporated into foundational methods [4] that have shaped our understanding of the functional elements in the genome. It also underpins methods for identifying and cataloging cancer driver genes [5, 6] that serve as references for biomedical research and cancer therapeutics [7].

In the past 15 years, a string of high-profile and hotly debated studies has provided evidence that challenges the neutrality assumption. Research from a range of model organisms has suggested that at least some synonymous mutations in some genes might have a distribution of fitness effects that is like non-synonymous mutations [8–10]. Experimental evidence has been found for both deleterious [9, 11] and adaptive [12, 13] fitness changes. In parallel, computational analyses have increasingly implicated synonymous mutations in human disease, including cancer-associated variation [14, 15], as well as proposed effects on microRNA-mediated regulation [16].

In 2022, a controversy arose from a study claiming that 70% of synonymous mutations in 21 essential yeast genes are non-neutral, a proportion comparable to nonsynonymous mutations [10]. This surprising finding was challenged due to methodological concerns [17]. In response, the authors published a follow-up study with more appropriately controlled experimental data [11] and found they had overestimated the magnitude of the fitness effects but defended their conclusion that most synonymous mutations are non-neutral [10, 11]. Because violating the assumption of synonymous neutrality would call much of evolutionary biology, bioinformatics, and disease genetics into question, and because these studies have relied on non-human model systems, a direct experimental interrogation of the fitness effects of synonymous mutations in human cells within the native genomic context is warranted. Recent prime editing experiments in human cells have begun to address this gap [18], and our study aims to contribute to this effort. We note at the outset that our study measures cellular fitness via proliferation in cell culture; we do not directly assess organismal fitness, developmental phenotypes, or tissue-specific contexts.

## Materials and methods

### Dataset

The K562 gene effect table, common essential gene list, common non-essential gene lists, and K562 transcript expression file were downloaded from DepMap 22Q2 (https://depmap.org/portal/data_page/?tab=allData). Canonical transcripts were retrieved from the MANE (Matched Annotation from NCBI and EBI) Project (https://www.ncbi.nlm.nih.gov/refseq/MANE/). Gene and transcript annotation were retrieved from GenCODE release 38 (https://www.gencodegenes.org/human/release_38.html). Protein-coding gene names across the whole genome were retrieved from BioMart (https://useast.ensembl.org/info/data/biomart/index.html).

### Essential gene selection

For essential genes, we selected genes in K562 that are < 0.01 quantile of the gene effect table and overlap with the common essential genes, resulting in 128 candidate genes (Supplementary Table S1). For non-essential genes, we selected genes with a gene effect score between –0.01 and 0.01, intersecting with the common non-essential genes, resulting in 41 non-essential genes (Supplementary Table S2).

### sgRNA library design

The selected essential gene list was used to run CHOPCHOP locally with a setup based on GRCh38 Genome Assembly. All possible single-guide RNAs (sgRNAs), defined as CRISPR targeting sequences that direct Cas9 to specific genomic sites, with NG PAM in the coding region and padding size 0, were returned. An updated setup for the Mac M1 Pro chip and the script can be found on GitHub (https://github.com/ryy1221/synSg).

To filter the unqualified sgRNAs, first, sgRNAs that can bind off-target sequences with fewer than two mismatches were filtered out. Then, sgRNAs overlapping with an intron–exon boundary were filtered out. For the MANE canonical transcript of each gene, the 15 bp base editing window (defined as –3 to +12 positions of the protospacer binding sequence, where 1 is the most PAM-distal position, and positions 21–23 are the PAM) was identified, and all possible edits within the window were predicted. To avoid confounding effects from canonical splicing, all sgRNAs targeting positions within ±2 base pairs of the intron–exon junction were excluded. The amino-acid alteration consequences of all possible edits were then predicted based on the hg38 annotation file.

For each gene (both essential and non-essential), the categories of sgRNAs were defined as:

1. Synonymous: All possible edits within the window generate synonymous mutations.
2. Missense: ≥1 edits within the window generate missense mutations.
3. Stop: ≥1 edits within the window generate nonsense mutations.
4. Empty Window: No edits were predicted in the target region.

We order the mutation types as Nonsense > Missense > Silent. Guides containing multiple mutation types were categorized by the most severe mutation type. All qualified synonymous sgRNAs were kept in the library.

To maintain a stringent definition of synonymous mutations, our analysis focused on synonymous mutations that do not overlap canonical splice sites. Specifically, we excluded positions within ±2 nucleotides of intron–exon junctions harboring the conserved AG–GU splice site motif. A minority of sgRNAs [4.35% in the ABE library (93/2 134) and 5.64% in the CBE library (199/3 528)] target sites within 8 base pairs of exon–intron boundaries. Our approach does not eliminate potential effects mediated by exonic splicing regulatory elements, such as enhancers or silencers (ESEs or ESSs).

For missense controls, we designed 599 sgRNAs targeting 878 potential missense mutations distributed across the same essential genes included in the synonymous library. This sample size is sufficient to detect missense hit rates down to ∼1% with narrow confidence intervals (0.5%–2.2%). While the number of synonymous sites far exceeds missense sites, the goal of the missense set was not to provide exhaustive coverage but to establish a robust benchmark for comparison against synonymous edits in the precise gene set the synonymous edits were drawn from.

Base editors have an “editing window,” a small range of nucleotides surrounding the target site where editing efficiency is maximized. When a sgRNA targets multiple codons, especially if these codons are spaced apart, it’s unlikely that all will fall within these high-efficiency windows. Therefore, for sgRNAs predicted to cause three or more mutations, we conservatively capped the total countable mutations at two for estimating the putative number of mutations introduced. This is a conservative estimation of mutation numbers given the validated NGS mutations.

### sgRNA and base editor vectors

The sgRNA libraries were cloned into the lentiCRISPR-v2-HygR-mCherry vector (Addgene #104991). For single sgRNA validation, individual sgRNAs were cloned into the lenti-ccdB-Dang-Hyg-sgGFP vector (Addgene #235047). The base editors used were ABE8e-SpG-Puro (Addgene #235044) for the ABE screen and CBEd-SpG-UGI-Puro (Addgene #235045) for the CBE screen. The constructed deaminase-null control, Ghost Editor, was nSpG-UGI-Puro (Addgene #235046).

### sgRNA library cloning

The final oligo sequences were attached with BsmBI sites and PCR primers following a modified version of the Doench Golden Gate cloning protocol. Every two sgRNAs were combined into one oligo, following the example:

ForwardPrimerTCTCACACC-sgRNA1-TCCGTTTCGAGACGCGTCTCACACC-reverse complement of sgRNA2-AGTTTCGAGA-ReversePrimer

An extra G was added to the start of the sgRNA if the sgRNA starts with C or T for enzyme digestion efficiency.

Primers for the ABE library:

- Forward Primer: GGGATCACTTACTACTTCCG
- Reverse Primer: GGTACCCTATCACTTAACCG

Primers for the CBE library:

- Forward Primer: CACTAGAGTAATCGCTACCG
- Reverse Primer: GGGCTACTAGACATAACTCG

Primers for the Dual iSilence library:

- Forward Primer: CCCTCTAGTCTTTCCAATCG
- Reverse Primer: GCCTGATATCACTCCTATCG

The libraries were synthesized by TwistBioscience.

The sgRNAs were amplified using KOD Hot Start DNA Polymerase (Sigma, 71086-3) and ligated (NEB, R0734S) to predigested lentiCRISPR-v2-HygR-mCherry vector via Golden Gate cloning.

### sgRNA library transformation

One day before electroporation, inoculate 10-beta competent cells (NEB, C3019H) in 200 ml media (20 g Tryptone/l, 10 g Yeast Extract/l). After the cell reached OD ∼ 0.5–0.6, cells were collected and washed in 10% glycerol three times (2500 × *g*, 15 min). Cells were resuspended in 10% glycerol after three washes. Per electroporation reaction, 10 µl ligation product was electroporated into 400 µl prepared 10-beta electrocompetent cells using Eppendorf Eporator (P2, 2500V). The electroporated cells were recovered at 37°C with 1 ml SOC for an hour and transferred to 200 LB media at 30°C for 16 h with 100 µg/ml carbenicillin. Electroporation efficiency was determined by plating 100× and 1000× dilutions on carbenicillin plates after recovery to ensure a library coverage of at least 500×.

### sgRNA library transfection and infection

Twenty-four hours before transfection, HEK293T cells were seeded in 10 cm dishes at a density of 0.5 × 10^6^ per ml in 10 ml of DMEM + 10% FBS. sgRNAs were transfected into HEK293T cells on day 1 with Gen2 lentiviral vector using calcium phosphate. Five 10 cm dishes of HEK293T cells were transfected with a total of 20 µg sgRNA library plasmid and 20 µg lentiviral v2 helper plasmids per plate. Media were changed 6 h after transfection and again on the second day. The viral media were collected 2 days after transfection, and at least 14M K562 and Jurkat cells were infected with 1 ml viral media for 5M cells, ensuring infection efficiency Multiplicity of Infection (MOI) < 1 and sgRNA coverage > 500×. Five days after infection, % mCherry positive cells were monitored and selected in 200 µg/ml hygromycin for another 7 days. Concurrently, 20 µg ABE8e, CBEd, and nSpG plasmids were transfected into HEK293T cells with 20 µg lentiv2 vectors, respectively to generate base editor viral media.

The day after hygromycin selection ended was set as D0, and at least 15M cells were collected as the baseline. On the same day, sgRNA-infected and selected cells were infected with base editors. Five days after infection, the cells were selected with puromycin (1 mg/ml for Jurkat, 1.5 mg/ml for K562). For the ABE arm, both cell lines were selected for 7 days, and cells were collected on Day 12. For the CBE arm, cells were collected on Day 12. K562 CBE arms were cultured for another 14 days and collected again on Day 26.

### Library preparation for sequencing

At least 10M cells were harvested for each library in each condition. Genomic DNA was extracted using phenol-chloroform [19] following cell lysis. For PCR amplification, staggered PCR was used. Detailed PCR primers can be found in the Supplementary Note. gDNA was divided into 100 µl reactions such that each well had at most 10 µg gDNA. PCR amplification was performed with Ex Taq (TaKaRa, RR001A), as described in previous protocol conditions and volumes [20]. PCR amplicons were extracted using gel extraction (OMEGA, D2500) and quantified by Qubit. Amplicons of sgRNA libraries were submitted to Novogene for PE150 sequencing.

### Single sgRNA validation experiment and sequencing

All candidate sgRNA sense and antisense oligos with BsmBI sites were ordered from IDT. Oligonucleotides were designed following the format *CACC–sense sgRNA* for the sense oligo and *AAAC–reverse-complement sgRNA* for the antisense oligo. All sgRNAs were identical to those used in the pooled screen and are listed in Supplementary Tables S3 and S4. Oligos were annealed and cloned into lentiCRISPR-v2-HygR-GFP Vector. K562 cells were cultured and infected with base editor plasmids used in the screen. Three days after infection, the infected cells underwent puromycin selection for base editor-containing K562 strains. Single sgRNA plasmids were then transfected with lentiviral packaging plasmids. The base editor-containing K562 cells were infected with single sgRNA plasmids. Five days after infection, the GFP-positive cells were tracked using flow cytometry for 12 days until Day 17. The expanded set of sgRNA was carried out in two replicates for 6 days.

At least 1M cells were collected at the start and end of the tracking period. gDNA was extracted using the NEB Monarch gDNA extraction kit (NEB, T1120S). Amplicons were amplified using Takara Bio Ex Taq polymerase, with primers that have standard Illumina adapters attached. The PCR products were then loaded on a gel and purified by gel extraction. Sequencing was performed by Genewiz Amplicon-EZ. To analyze genome editing outcomes, we used CRISPResso to process sequencing data from edited cell populations. Paired-end FASTQ files were provided as input, along with the reference amplicon sequence and an assigned amplicon name. Default parameters were used. The output directory contained quantifications of editing efficiency and mutation types, enabling downstream analysis of genome editing events. We applied filtering criteria to retain only sequences with at least 1% of mapped reads and a minimum of 100 read counts. Sequence modifications were identified by aligning observed sequences to reference amplicons and pinpointing nucleotide changes. Genomic positions of mutations were determined relative to the amplicon start position. We then compared observed mutations to predicted editing sites derived from prior computational analyses of sgRNA targets. To assess consistency across experimental replicates, we examined sequence intersections and calculated allele frequencies at two timepoints (Day 5 and Day 17).

### Library sequencing analysis

Guide sequences were extracted from the FASTA file of the library sequencing. First, the U6 promoter and gRNA scaffold TTGTGGAAAGGACGAAACACC…GTTTCAGAGCTATGCT was trimmed using cutadapt. High-quality trimmed reads were then aligned against the FASTA file of all sgRNA sequences using bowtie [21]. The aligned reads were processed using custom code (available on GitHub) to obtain the read counts of sgRNAs.

Before proceeding to correlation coefficient calculation, read counts were first normalized to log2reads per million using:


\begin{eqnarray*}
&& {\boldsymbol{log}} - {\boldsymbol{normalized}}{\mathrm{\ }}{\boldsymbol{reads}}{\mathrm{\ }}{\boldsymbol{per}}{\mathrm{\ }}{\boldsymbol{million}} = {\boldsymbol{log}}2\\&& \left( {\frac{{{\boldsymbol{reads}}{\mathrm{\ }}{\boldsymbol{per}}{\mathrm{\ }}{\boldsymbol{guid}}}}{{{\boldsymbol{total}}{\mathrm{\ }}{\boldsymbol{reads}}{\mathrm{\ }}{\boldsymbol{per}}{\mathrm{\ }}{\boldsymbol{condition}}}}{\boldsymbol{X}}{\mathrm{\ }}1,000,000 + 1} \right)
\end{eqnarray*}

DESeq2 was installed in a separate conda environment following the instructions: https://pydeseq2.readthedocs.io/en/latest/. DESeq2 uses the Benjamini–Hochberg method for multiple hypothesis testing. We then used *PyDESeq2* to determine sgRNA log2foldchange depletion. All dropout comparisons were made to the D0 baseline library.

### SgRNA editing efficiency estimation

We utilized the *BE-Hive* package [22] to predict the editing efficiencies of sgRNAs in mouse embryonic stem (mES) cells, the only suspension cell line in their dataset that closely resembles K562 and Jurkat cells. ABE sgRNA efficiencies were predicted using the ABE model, while CBE sgRNA efficiencies were predicted using the BE4 model. The BE-Hive tool generates a logit score for each sgRNA, representing its propensity for base editing. This score is then transformed into a fraction—corresponding to the proportion of sequenced reads exhibiting base editing activity—using a logistic function: ${\boldsymbol{Base}}\ {\boldsymbol{editing}}\ {\boldsymbol{Activity}} = \ \frac{1}{{1 + {{\bf exp}} ({ - ( {{\boldsymbol{logit}}\ {\boldsymbol{score}}\ {\boldsymbol{X}}\ {\boldsymbol{std}} + {\boldsymbol{m}}e{\boldsymbol{an}}} )} )}}$. According to the iSilence control, we use an average editing efficiency of 30% to perform the transformation (default standard deviation = 2). For each sgRNA, we calculated the adjusted fraction of edited reads based on this efficiency. sgRNAs with reading counts falling below two standard deviations from the mean were excluded from further analysis.

### Estimation of fold difference and confidence interval

Our goal is to estimate the fold difference in the proportion of sgRNAs that produce measurable fitness effects between synonymous and missense mutations. We define the probability of a mutation having functional effect as π. π conceptually corresponds to the hit rate observed in the screen and is a naïve estimate that reflects the apparent frequency of functional variants. However, it does not account for assay sensitivity or specificity. Therefore, to better approximate the experimental process and incorporate validation data, we developed a likelihood-based framework that integrates information from both the pooled screen (benchmarked against positive and negative controls) and the single-sgRNA validation assays (which provide confirmed true and false positives). The model estimates the underlying probability of functional effects for synonymous (${{{\boldsymbol{\pi }}}_{{\boldsymbol{synonymous}}}}$) and missense (${{{\boldsymbol{\pi }}}_{{\boldsymbol{missense}}}}$) sgRNAs, respectively, enabling quantification of the fold difference in their functional impact.

We used DESeq2 Wald statistic ${\boldsymbol{s}} = \ {\boldsymbol{LFC}}\ /\ {\boldsymbol{SE}},$ where LFC and SE are DESeq2 Log2Foldchange and the estimated standard error of LFC. The statistics is a normalized measure of sgRNA depletion/enrichment signal to noise. We pooled sgRNA-level signals across K562 and Jurkat, and across ABE and CBE (D14 for K562) screens.

We model the observed sgRNA-level statistics as a mixture of two reference distributions: ${{{\boldsymbol{f}}}_0}$​ for negative-control guides representing null (non-functional) effects, and ${{{\boldsymbol{f}}}_1}$​ for positive-control guides representing true functional effects.

For negative-control guides (non-targeting and empty-window), represented by ${{{\boldsymbol{s}}}_{\boldsymbol{k}}}$​, the distribution of was modeled as a single Gaussian:


\begin{eqnarray*}
{{{\boldsymbol{f}}}_0}\left( {{{{\boldsymbol{s}}}_{\boldsymbol{k}}};{{{\boldsymbol{\mu }}}_0},{{{\boldsymbol{\sigma }}}_0}} \right)\ = \ {\boldsymbol{N}}\left( {{{{\boldsymbol{s}}}_{\boldsymbol{k}}};{{{\boldsymbol{\mu }}}_0},{{{\boldsymbol{\sigma }}}_0}} \right)
\end{eqnarray*}

For positive-control guides (iSilence), represented by ${{{\boldsymbol{s}}}_{\boldsymbol{l}}}$, we accounted for the presence of known false negatives (i.e. sgRNAs designed for essential sites that do not always produce strong depletion phenotypes) by modeling the positive-control distribution as a two-component Gaussian mixture. One component captures strong depletion effects and the other represents weak or near-neutral signals:


\begin{eqnarray*}
{{{\boldsymbol{f}}}_1}\left( {{{{\boldsymbol{s}}}_{\boldsymbol{l}}};{{{\boldsymbol{\mu }}}_1},{{{\boldsymbol{\sigma }}}_1},{{{\boldsymbol{\mu }}}_2},{{{\boldsymbol{\sigma }}}_2}} \right)\ = \ {{{\boldsymbol{w}}}_1}{\boldsymbol{N}}\left( {{{{\boldsymbol{s}}}_{\boldsymbol{l}}};{{{\boldsymbol{\mu }}}_1},{{{\boldsymbol{\sigma }}}_1}} \right)\ + \ {{{\boldsymbol{w}}}_2}{\boldsymbol{N}}\left( {{{{\boldsymbol{s}}}_{\boldsymbol{l}}};{{{\boldsymbol{\mu }}}_2},{{{\boldsymbol{\sigma }}}_2}} \right)
\end{eqnarray*}

, where component weights ${{{\boldsymbol{w}}}_1}$and ${{{\boldsymbol{w}}}_2}\ $sum to 1, and all parameters were estimated by expectation–maximization (EM) using the *sklearn-GuassianMixture* implementation.

Therefore, for a screened guide with score ${{{\boldsymbol{s}}}_{\boldsymbol{i}}}$​, the likelihood of observing the data given the model is expressed as a mixture of the negative and positive distribution:


\begin{eqnarray*}
{\boldsymbol{P}}\left( {{{{\boldsymbol{s}}}_{\boldsymbol{i}}}^{{\boldsymbol{screen}}};{\boldsymbol{\pi }},{{{\boldsymbol{f}}}_0},{{{\boldsymbol{f}}}_1}} \right)\ &=& \ {\boldsymbol{\pi }}{{{\boldsymbol{f}}}_1}\left( {{{{\boldsymbol{s}}}_{\boldsymbol{l}}};{{{\boldsymbol{\mu }}}_1},{{{\boldsymbol{\sigma }}}_1},{{{\boldsymbol{\mu }}}_2},{{{\boldsymbol{\sigma }}}_2}} \right)\ \\&+& \ \left( {1 - {\boldsymbol{\pi }}} \right){{{\boldsymbol{f}}}_0}\left( {{{{\boldsymbol{s}}}_{\boldsymbol{k}}};{{{\boldsymbol{\mu }}}_0},{{{\boldsymbol{\sigma }}}_0}} \right)
\end{eqnarray*}

Across all screened sgRNAs, the joint likelihood of the proportion of functional effects given the data is


\begin{eqnarray*}
&& {{{\boldsymbol{L}}}_{{\boldsymbol{screen}}}}({{\bf \pi }}|\{ {{{\boldsymbol{s}}}_{\boldsymbol{i}}}\} _{{\boldsymbol{i}} = 1}^{{{{\boldsymbol{N}}}_{{\boldsymbol{screen}}}}},{{{\boldsymbol{f}}}_0},{{{\boldsymbol{f}}}_1}){\mathrm{\ }} =\\&& {\mathrm{\ }}\mathop \prod \limits_{{\boldsymbol{i}} = 1} \ \left[ {{\boldsymbol{\pi }}{{{\boldsymbol{f}}}_1}\left( {{{{\boldsymbol{s}}}_{\boldsymbol{i}}}^{{\boldsymbol{screen}}}} \right)\ + \ \left( {1 - {\boldsymbol{\pi }}} \right){{{\boldsymbol{f}}}_0}\left( {{{{\boldsymbol{s}}}_{\boldsymbol{i}}}^{{\boldsymbol{screen}}}} \right)} \right]
\end{eqnarray*}

For guides that were individually validated, each outcome provides direct evidence of being functional; therefore:

$${\boldsymbol{P}}({{{\boldsymbol{s}}}_{\boldsymbol{j}}}^{{\boldsymbol{validated}}},{\mathrm{\ \ }}{{{\boldsymbol{y}}}_{\boldsymbol{j}}}|{\boldsymbol{\pi }},{{{\boldsymbol{f}}}_0},{{{\boldsymbol{f}}}_1}){\mathrm{\ }} = {\mathrm{\ }}\{ {\begin{array}{@{}*{1}{c}@{}} {\pi {{{\boldsymbol{f}}}_1}( {{{{\boldsymbol{s}}}_{\boldsymbol{j}}}^{{\boldsymbol{validated}}}} )\ {\mathrm{\ }}{{{\boldsymbol{y}}}_{\boldsymbol{j}}}\ = \ 1}\\ {( {1 - \pi } ){{{\boldsymbol{f}}}_0}({{{\boldsymbol{s}}}_{\boldsymbol{j}}}|{{{\boldsymbol{\mu }}}_0},{{{\boldsymbol{\sigma }}}_0})\ {\mathrm{\ }}{{{\boldsymbol{y}}}_{\boldsymbol{j}}}\ = \ 0} \end{array}}$$

Across all validated guides, the validated data likelihood is therefore:


\begin{eqnarray*}
&&{{{\boldsymbol{L}}}_{{\boldsymbol{validated}}}}({{\bf \pi }}|\{ {{{\boldsymbol{s}}}_{\boldsymbol{j}}},{{{\boldsymbol{y}}}_{\boldsymbol{j}}}\} _{{\boldsymbol{j}} = 1}^{{{{\boldsymbol{N}}}_{{\boldsymbol{validated}}}}},{{{\boldsymbol{f}}}_0},{{{\boldsymbol{f}}}_1})\ =\\&& \mathop \prod \limits_{\boldsymbol{j}} \ {{[{\boldsymbol{\pi }}{{{\boldsymbol{f}}}_1}({{{\boldsymbol{s}}}_{\boldsymbol{j}}}|{{{\boldsymbol{\mu }}}_1},{{{\boldsymbol{\sigma }}}_1},{{{\boldsymbol{\mu }}}_2},{{{\boldsymbol{\sigma }}}_2})]}^{{{{\boldsymbol{y}}}_{\boldsymbol{j}}}}}\ {{\left[ {\left( {1 - {\boldsymbol{\pi }}} \right){{{\boldsymbol{f}}}_0}\left( {{{{\boldsymbol{s}}}_{\boldsymbol{j}}}} \right)} \right]}^{\left( {1 - {{{\boldsymbol{y}}}_{\boldsymbol{j}}}} \right)}}
\end{eqnarray*}

We next combined the screened and validated likelihoods to obtain a joint likelihood for estimating the probability of functional effects:


\begin{eqnarray*}
{\boldsymbol{L}}({{\bf \pi }}|{{\bf \textit{data}}}){\mathrm{\ }} = {\mathrm{\ }}{{{\boldsymbol{L}}}_{{\boldsymbol{screen}}}}({{\bf \pi }}|{{\bf \textit{data}}}){\mathrm{\ }} \times {\mathrm{\ }}{{{\boldsymbol{L}}}_{{\boldsymbol{validated}}}}({{\bf \pi }}|{{\bf \textit{data}}})
\end{eqnarray*}

Because synonymous and missense sgRNAs exhibit systematically different magnitudes of depletion, we incorporated an effect-size adjustment into the likelihood. On average, missense candidates showed 8-fold greater depletion than synonymous candidates in the single-sgRNA validation assays, as measured by the relative reduction in GFP-positive cells. To reflect this stronger effect, we applied a proportionally larger weighting factor *c* to the posterior contribution of missense guides. This weighting ensures that the higher depletion magnitude of missense sgRNAs is appropriately represented in the inferred probability of functional effects. Accordingly,


\begin{eqnarray*}
{\boldsymbol{L}}({{{{\bf \pi }}}_{{\boldsymbol{missense}}}}|{{\bf \textit{data}}}){\mathrm{\ }} &=& {\mathrm{\ }}{{{\boldsymbol{L}}}_{{\boldsymbol{screen}}}}{{({{{{\bf \pi }}}_{{\boldsymbol{missense}}}}|{{\bf \textit{data}}})}^{\boldsymbol{c}}}{\mathrm{\ }} \\&& \times {\mathrm{\ }}{{{\boldsymbol{L}}}_{{\boldsymbol{validated}}}}({{{{\bf \pi }}}_{{\boldsymbol{missense}}}}|{{\bf \textit{data}}}){{\ }^{\boldsymbol{c}}}
\end{eqnarray*}

\begin{eqnarray*}
{\boldsymbol{L}}({{{{\bf \pi }}}_{{\boldsymbol{synonymous}}}}|{{\bf \textit{data}}}){\mathrm{\ }} &=& {\mathrm{\ }}{{{\boldsymbol{L}}}_{{\boldsymbol{screen}}}}({{{{\bf \pi }}}_{{\boldsymbol{synonymous}}}}|{{\bf \textit{data}}}){\mathrm{\ }} \\&& \times {\mathrm{\ }}{{{\boldsymbol{L}}}_{v{\boldsymbol{alidated}}}}({{{{\bf \pi }}}_{{\boldsymbol{synonymous}}}}|{{\bf \textit{data}}})
\end{eqnarray*}

The likelihood was evaluated numerically over a grid of π values (100 000 points) and normalized.

To quantify the relative frequency of functional effects between mutation classes, we estimated the distribution of the fold difference ${\boldsymbol{r}} = \frac{{{{{{\bf \pi }}}_{{\mathrm{missense}}}}}}{{{{{{\bf \pi }}}_{{\mathrm{synonymous}}}}}}$. On average, synonymous and missense sgRNAs generate a similar number of edited alleles per guide, making the sgRNA-level hit rate an appropriate basis for estimating fold difference. In addition, both categories are restricted to SNV-accessible changes in base-editing experiment. For each category, we generated 10 000 random draws of ${{{{\bf \pi }}}_{{\boldsymbol{missense}}}}$ and ${{{{\bf \pi }}}_{{\boldsymbol{synonymous}}}}$, calculated the ratio, and their ratios were used to estimate the empirical distribution of *r*. The median and 95% credible interval of *r* were then reported as the inferred fold difference in the probability of functional effects between missense and synonymous sgRNAs, integrating evidence from both the pooled screen and validation experiments.

## Results

### Framework for isolating synonymous variant effects in pooled screens

Several recent large-scale genome editing studies report that synonymous mutations exhibit extensive non-negligible fitness effects. For example, 21% [23] of synonymous mutations in the BRCA1 gene appear to be loss-of-function variants as determined via a deep mutagenesis assay. In contrast, a tiling base-editing assay estimated this same proportion to be 1.2% [24]. Similarly, base editing screens targeting “core” fitness [25, 26] genes reported that between 5.2% [27] (in 7 genes) and 11.5% [28] (in 10 genes) of sgRNAs directed at synonymous sites created fitness defects that caused the frequency of synonymous editing sgRNAs to deplete in the population. This wide range of reported values (1.2%–21%) likely stems from the fact that these studies’ primary focus was on characterizing non-synonymous mutation effects. It is therefore possible that the observed significant synonymous sgRNA hits are less reliable than the non-synonymous results because the studies did not explicitly attempt to provide controls for the synonymous hits or engage in further experimental validation of synonymous mutations.

**Figure 1.**
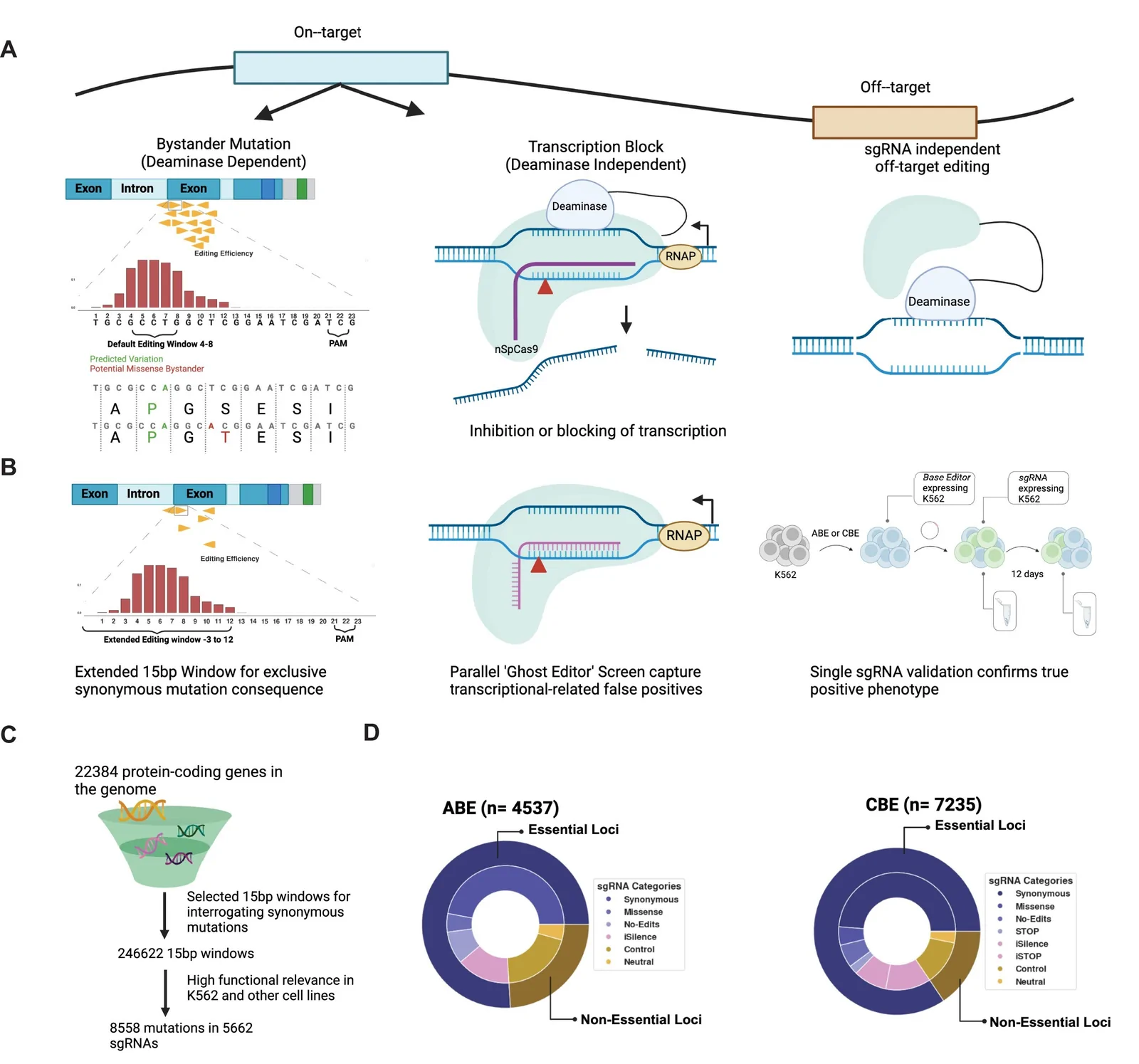
Minimizing false positives in base editor screens of synonymous mutations. (**A**) Key sources of false positives in pooled sgRNA screens: (i) Bystander mutations (deaminase-dependent): Base editing can introduce bystander mutations within the default core editing window (typically 4–8 bp), resulting in unintended missense mutations. This confounds the intended synonymous mutations, leading to false positive phenotypes. (ii) Transcriptional block (deaminase-independent): The binding of nCas9 to single-stranded DNA can inhibit transcription initiation or elongation, potentially reducing gene expression. This deaminase-independent effect can mimic gene knockdown, producing false positive depletion phenotypes. (iii) Off-target editing: sgRNAs may bind to off-target sites in the genome, leading to unintended base editing at functional loci. This confounding variation can falsely suggest gene essentiality or an altered phenotype. (**B**) Strategies to isolate the effects of synonymous mutations: (i) extended editing window: By expanding the base editing window from the default 5 to 15 bp, sgRNA designs can reduce the likelihood of bystander missense mutations, increasing the chance of inducing pure synonymous changes. (ii) “Ghost” editor control: A parallel screen using a deaminase-null base editor (referred to as the “Ghost Editor”) should be performed with the same sgRNA library. This controls for transcriptional block effects, enabling the identification of transcription-related false positives. Single sgRNA validation: Hits from pooled screens should undergo validation with individual sgRNAs to confirm true positive phenotypes and distinguish them from false positives arising from off-target or bystander effects. (**C**) Diagram of selecting sgRNA for an unbiased screen. A total of 128 target genes were selected for screening. (**D**) Composition of ABE and CBE sgRNA libraries. The ABE library consists of 4537 sgRNAs, including synonymous sgRNAs, missense sgRNAs, sgRNAs designed to make no edits in the same essential gene, and positive controls such as sgRNAs targeting start codons (iSilence) in essential loci. In non-essential loci, neutral sgRNAs target K562-neutral genes, and control sgRNAs include non-targeting sgRNAs. The CBE library consists of 7235 sgRNAs. In addition to the categories in the ABE library, the CBE library includes sgRNAs that induce stop codons in essential genes (iSTOP) and an iSTOP positive control library.

To directly address this limitation, we designed a new base editing study to interrogate the fitness consequences of specific synonymous mutations at the endogenous genomic locus in human cells. In doing so, we identified three potential sources of false positives (Fig. 1A). First, unintended non-synonymous edits due to bystander mutations beyond a base editor’s core editing window. Most base editing studies report only the highest probability 5-nt “core” editing window within the sgRNA sequence. This region, typically spanning nucleotide positions 4–8 relative to the protospacer adjacent motif (PAM), exhibits the highest deaminase activity and editing efficiency [25, 26]; however, editing activity occurs outside this core window in at least 20% of cases [29]. This means that many guides that are labeled as “synonymous” guides in existing studies make only synonymous mutations in the smaller 5-nt high-probability editing window, but, have a significant potential to make non-synonymous mutations just outside this window. Second, Cas9-independent off-target effects caused by base editors acting on single-stranded DNA could produce mutations randomly in the genome [30, 31]. Even though the rate of such an effect could be estimated by safe harbor targeting guides [28], the resulting false positive effect would still appear in a pooled screen. Finally, there are potentially overlooked editing independent effects from Cas9 nickase activity disrupting essential gene expression [32]. Cas9, for example, can repress transcription by obstructing initiation or elongation when sgRNAs target regions are near promoters [32, 33]. The extent of this repression is influenced by factors such as the sgRNA’s position relative to the promoter, guide composition, chromatin state, and the concentration of the Cas9/sgRNA complex [34].

To address these issues, we implemented a four-pronged validation framework not used in previous studies (Fig. 1B): (i) we increased the base editing window to 15 bp to ensure that our “synonymous” sgRNAs produced exclusively synonymous mutations across the entire editing window, not just the 80% of edits in a 5 bp window; (ii) We used a “ghost editor control,” which is a deaminase-null base editor, thus any sgRNA hits from the library in this deaminase deficient screen are editing-independent false positives because no mutations are made. (iii) We carried out independent infections of the library to control for Cas9-independent editing effects. (iv) We validated significant hits from the pooled screen with single sgRNA assays followed by direct sequencing to detect any off-target edits.

To establish a large sgRNA library to systematically investigate the fitness effects of synonymous mutations across the human genome, we aimed to maximize the number of “synonymous mutation only” sgRNAs within highly essential genes across multiple cell lines using the efficient and specific NG-PAM base editors, ABE8e [35] and CBEd [36, 37]. Starting with 22 384 protein-coding genes in the genome, we applied a data-driven approach to identify regions of interest. Specifically, we identified all 15 bp windows within coding regions that were devoid of the possibility of non-synonymous edits, thereby focusing on regions where only synonymous mutations could be interrogated for their potential functional impacts. This resulted in 246 622 windows that we could target (115 401 targeted by the ABE base editor and 131 221 targeted by the CBE editor). Next, we restricted this set to 128 genes that were highly essential in both of our cell models by DepMap [25] gene effect score and broadly essential across diverse cellular contexts and created a sgRNA library that can target 5662 windows (2134 in ABE and 3528 in CBE) with the potential to introduce 8558 putative synonymous mutations (2964 targeted by ABE and 5594 targeted by CBE; Supplementary Tables S1–S8). There are more putative synonymous mutations than sgRNAs because a single guide has the potential to create multiple mutations within the target window (Fig. 1C).

To ensure a robust experimental design, we incorporated rigorous controls into the sgRNA library. Negative controls included sgRNAs with no predicted edits within the exact same gene (*n *= 407 in ABE, *n *= 458 in CBE), non-targeting sgRNAs (*n *= 613 in ABE and CBE), sgRNAs targeting safe harbor sites (*n *= 282 in ABE and CBE), and neutral sgRNAs targeting non-essential genes (*n *= 196 in ABE, *n *= 231 in CBE). Positive controls included sgRNAs that create missense mutations in the same essential genes (*n *= 232 in ABE, *n *= 367 in CBE), sgRNAs that create premature stop codons in the same essential genes (*n *= 181 in CBE), sgRNAs designed to disrupt start codons (known as iSilence, *n *= 673) [38] in both the ABE and CBE libraries, and previously published iSTOP sgRNAs (*n *= 902) [39] in the CBE library. The final library contains 4537 and 7235 sgRNAs, respectively, for the ABE and CBE libraries, which are composed of controls, synonymous and missense mutation-causing sgRNAs (Fig. 1D, see Supplementary Note for Library Composition, Supplementary Tables S3 and S4).

To mitigate base editor-induced off-target effects and cell line-specific effects, we independently transduced three replicates of K562 and Jurkat cells, with the sgRNA library on a separate vector (denoted D0). Following the library introduction, replicates were again independently infected with ABE8e, CBEd, or the deaminase-null Ghost Editor. After infection and selection, the first endpoint was day 12 (D12) for both ABE and CBE screens in K562 and Jurkat cells (Supplementary Fig. S1). Because cytosine base editing positive controls accumulate true positive hits more slowly than adenine base editing in pooled assays (Supplementary Table S14), we extended the K562 CBE screen by an additional 14 days (to Day 26) to ensure sufficient time to maximize sensitivity for detecting CBE hits. Indeed, we observed that CBE hits continued to rise between Day 12 and Day 26 (Supplementary Figs S3 and S4). Cells were then harvested for next-generation sequencing (NGS) to assess sgRNA representation at the baseline and Day 12 or Day 26 endpoint. Differential read counts were analyzed using DESeq2 [40].

### Fitness effects of synonymous variants in essential genes are extremely rare in human cell lines

Positive control libraries (iSilence), as expected, exhibited a high concordance of normalized log2 fold changes (L2FC) across biological replicates (Supplementary Figs S2–S4), with Pearson’s *r* = 0.87 in Jurkat and 0.83 in K562 for ABE8e, and Pearson’s *r* = 0.64 in K562 and 0.79 in Jurkat, confirming consistency across replicates. We observed ABE8e iSilence hit rates (adjusted *P*-value < 0.05) of 33.2% (Jurkat) and 37.0%(K562) of total positive control sgRNAs. We observed negative control sgRNAs hit rates ranging from 0% to 4% (Supplementary Figs S2–S4). These results are consistent with the prior literature and indicate a successful screen [28]. An analysis of the AUC for hit calling across positive and negative controls yielded a low false positive rate with medium sensitivity (Supplementary Fig. S13).

The strong performance of the screen in our positive and negative control guide sets gave us confidence that we could analyze the missense and the synonymous hits from the screen. To do this, we looked for guides that were significantly enriched or depleted on Day 12 and/or Day 26. We filtered out guides that were also hit in our ghost editor screen (Supplementary Note, Supplementary Fig. S5) as they are likely false positives. We initially applied an adjusted *P*-value threshold of 0.05 for identifying significant synonymous sgRNAs (referred to as the stringent set). Because synonymous mutations appeared to generally yield weaker depletion signals in pooled screens, we also adopted a more permissive threshold (adjusted *P* ≤ 0.2) to increase our ability to identify rare true positives and reduce the confidence intervals of our measurements (this constituted “expanded set” data added later, and is shown in Supplementary Fig. S11). The additional sgRNAs in the expanded set were also evaluated using single sgRNA competition assays. This added 16 additional synonymous sgRNAs (5 ABE, 11 CBE).

**Figure 2.**
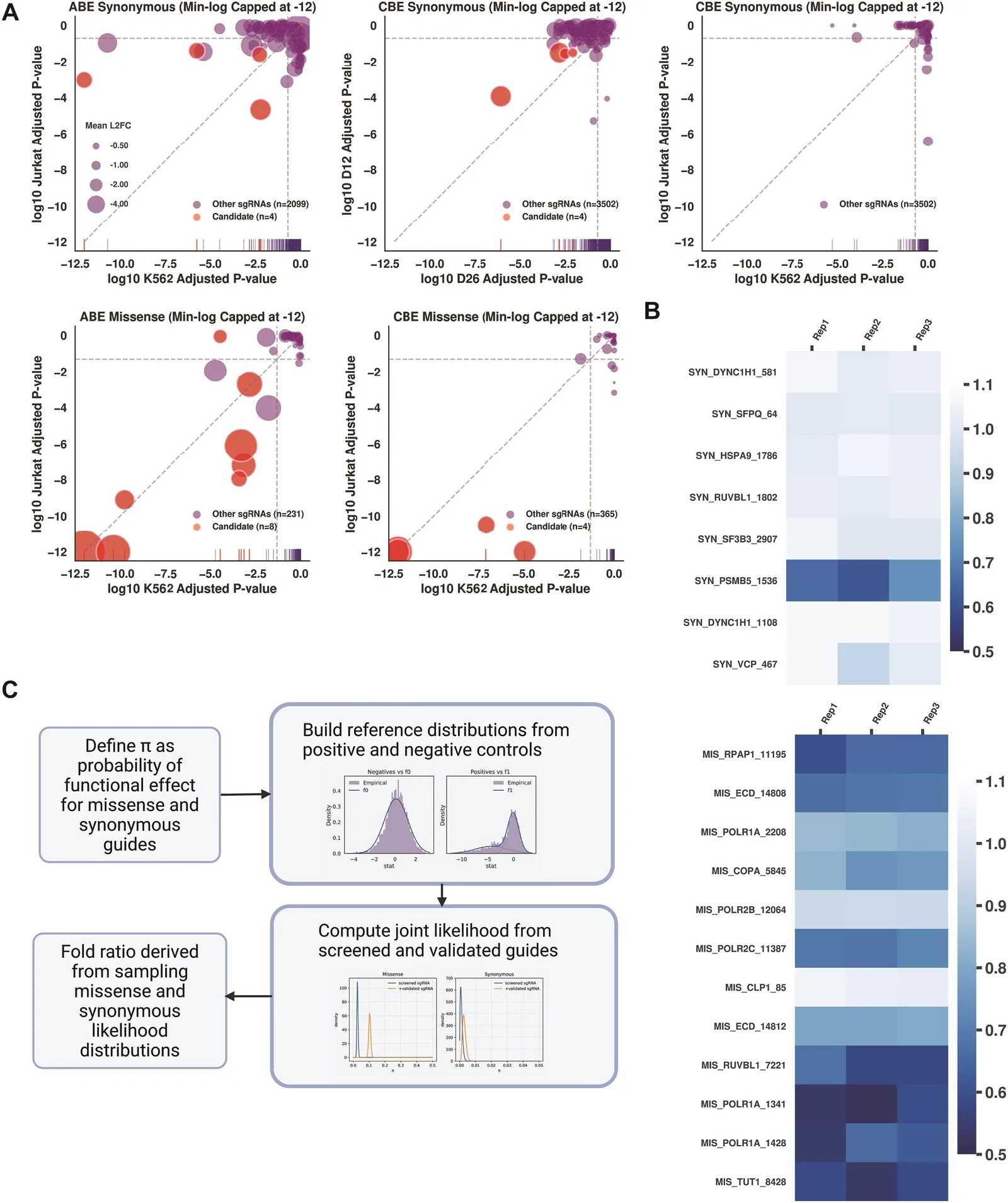
Experimental validation of synonymous sgRNA hits. (**A**) Scatter plots compare the significance of sgRNA depletion across independent conditions. For ABE screens, results in K562 versus Jurkat cells are shown for both synonymous and missense-targeting sgRNAs. For CBE screens, results are shown for missense-targeting sgRNAs (K562 versus Jurkat) and for synonymous-targeting sgRNAs (Day 26 versus Day 12 in K562, since no overlapping candidates were found between cell lines). Each dot represents an sgRNA; Highlighted dots indicate sgRNAs that scored as significant candidates in both conditions. Dot size reflects the mean log2 fold change, and *P*-values are capped at –log10(padj) = 12 for visualization. (**B**) Heatmap showing the percentage of GFP + cells over time in competition assays for candidate sgRNAs. Each row represents an independent sgRNA replicate, and colors reflect the ratio of GFP + cells on Day 17 relative to Day 5. Lower D17 relative to D5 GFP+ ratio indicate stronger depletion. Of the eight synonymous sgRNAs tested, only one validated as a true positive, whereas 11 of 12 missense sgRNAs validated. (**C**) Positive-control (iSilence) and negative-control (empty-window and non-targeting) sgRNAs were used to fit the score distributions for functional (${{f}_1}$) and neutral (${{f}_0}$​) guides. Unlabeled sgRNAs from the pooled screen contributed a mixture-likelihood term, and single-sgRNA validation outcomes contributed a labeled-likelihood term weighted by the depletion magnitude (C). The combined likelihood was used to estimate the probability π that each mutation class produces a functional effect. Fold differences between synonymous and missense mutation classes were calculated from repeated sampling of the fitted distributions.

With these sgRNA sets in hand, we performed single sgRNA competition assays (mutant versus wild-type) across multiple time points for 24 synonymous sgRNAs (8 stringent hits in Fig. 2B + 16 expanded hits in Supplementary Figs S11 and S12) and missense sgRNA hits. To perform these experiments, we established K562 cell lines stably expressing base editors and monitored the proportion of GFP-tagged sgRNA-expressing cells in a partially transduced population to assess depletion.

We observed 1 out of 8 synonymous sgRNAs in the stringent set of hits (SYN_PSMB5_1536; Supplementary Fig. S6B) and 1 out of 16 in the expanded set (SYN_DDX54_1948; Supplementary Fig. S11A) showed strong GFP depletion, both confirmed by targeted sequencing (Supplementary Figs S7 and S11B, and Supplementary Table S9). In contrast, 11 of the 12 missense sgRNAs across both the ABE and CBE libraries exhibited significant depletion (Supplementary Figs S6 and S7, Supplementary Note). Altogether, our validation assays revealed a 91.6% true-positive rate (11/12) for missense sgRNAs but only 8.3% (2/24) for synonymous sgRNAs. This disparity does not reflect a limitation in the screening platform but the genuine rarity of guides that create synonymous mutations that measurably impact fitness in K562 and Jurkat cells.

To further confirm the authenticity of the validated synonymous hits, we performed whole-genome sequencing of cells edited with SYN_PSMB5_1536 to identify possible off-target events that might generate missense mutations (Supplementary Table S11). Two missense mutations in essential genes were detected in all replicates; however, neither showed depletion over time, indicating they arose in distinct subpopulations without fitness defects (Supplementary Note, Supplementary Table S10). In addition, all high-scoring off-target alignments for the SYN_DDX54_1948 mapped to non-essential or non-coding regions (Supplementary Table S16). These results further support that the validated synonymous edits represent true positive fitness-affecting mutations.

In addition, the PSMB5 synonymous mutation candidate (chr14:23 033 420 G > A) was predicted by SpliceAI to substantially alter splice-site strength (SpliceAI Δscore > 0.9; Pangolin splice gain/loss > 0.7), suggesting a high-confidence disruption of splicing, despite our efforts to exclude splice-altering mutations (Supplementary Table S15). However, a detailed mechanistic investigation of this variant is beyond the scope of this study, which focuses on quantifying the overall frequency and statistical properties of synonymous fitness effects across the genome.

### Estimation of the relative probability of synonymous fitness effects

We next sought to estimate the relative probability that a synonymous mutation produces a measurable fitness effect compared with a missense mutation. To do so, we constructed a likelihood-based mixture model that integrates information from both the pooled screen and single-sgRNA validation assays. Briefly, the distribution of DESeq2 Wald statistics for negative-control guides (expected to be neutral) was modeled as a single Gaussian (${{{\boldsymbol{f}}}_0}$​), and the distribution for positive-control guides (iSilence) was modeled as a two-component Gaussian mixture (${{{\boldsymbol{f}}}_1}$) to capture both strong and weak/no depletion effects. For each sgRNA, we estimated its probability π of belonging to the functional component ${{{\boldsymbol{f}}}_1}\ $by maximizing the joint likelihood of the pooled-screen statistics and the single-sgRNA validation results, incorporating a weighting factor proportional to the depletion magnitude observed in the validation assays. See method for detailed explanation.

From this model, we estimated that the fraction of synonymous guides with functional effects (${{{{\bf \pi }}}_{{\boldsymbol{synonymous}}}}$) was 0.0028 (95% credible interval: 0.0011–0.0053), whereas the fraction of non-synonymous guides with functional effects (${{{{\bf \pi }}}_{{\boldsymbol{missense}}}}$) was 0.1038 (95% credible interval: 0.0918–0.1165). To estimate uncertainty, we repeatedly sampled from the fitted distributions of ${{{\mathrm{\pi }}}_{\textit{missense}}}$​ and ${{{\mathrm{\pi }}}_{\textit{synonymous}}}$​ and calculated their ratio, yielding a median fold difference of 37.9 (95% confidence interval [CI], 22.16–81.48 (Fig. 2C)). Thus, missense mutations were measured to be 37.9× more likely to produce a fitness-altering effect than synonymous mutations.

While the likelihood ratio suggests that synonymous and missense mutations do not have similar effects on fitness, as suggested elsewhere [41], we also wanted to evaluate whether synonymous mutations tend to behave neutrally. To do this we compared the hit rate distributions between synonymous-targeting sgRNAs and multiple categories of negative control sgRNAs (non-targeting, safe harbor, and neutral-targeting). The interquartile range (IQR) of hit rates for negative control sgRNAs spans from 0.34% to 3.43%, with a median of 1.03%. Synonymous-targeting sgRNAs exhibited a similar median hit rate of 0.95%, with values ranging from 0.22% to 1.22%. Grouping negative control sgRNAs did not alter the observed similarity in distribution. When grouped, negative control sgRNAs showed an interquartile range of 0.26%–2.2%, which remains comparable to that of synonymous-targeting sgRNAs (Supplementary Table S12). These results show that, in aggregate, sgRNAs generating synonymous mutations display distributions of pooled fitness effects that are statistically indistinguishable from negative controls, indicating that synonymous mutations rarely produce detectable fitness effects under our experimental conditions.

In conclusion, this 37.9× fold difference stands in contrast to observations in yeast, where synonymous mutations have been reported to produce fitness effects at frequencies comparable to missense mutations [10, 11]. Our observed hit rate for synonymous-targeting sgRNAs (0.95%) is statistically indistinguishable from negative controls and consistent with recent prime editing results in human cells [18], suggesting that the pervasive synonymous fitness effects reported in yeast do not generalize to human cells under standard cell culture conditions.

## Discussion

Our study employed a new four-pronged approach to estimate the fitness effects of synonymous mutations in highly essential genes within their endogenous genomic context using base editing. We designed our library with stringent criteria, selecting 15-bp windows in essential genes that permit only synonymous edits (avoiding the alteration of splicing), thereby eliminating the possibility of confounding non-synonymous mutations or large changes in amino acid sequence from splice alterations. We included a novel deaminase-deficient editing control that filtered out the base edits that score in the absence of a functional editor, and we validated depletion phenotypes and base edits in independent validation assays that were not included in several other studies [27, 28]. The similar hit rates observed for synonymous and non-synonymous mutations in those studies may be attributable to the absence of such controls and a lack of follow-up 1-by-1 validation. Ultimately, while missense mutations define a robust and truly distinct functional distribution in our data (91.6% validated true-positive rate), the rare synonymous “hits” (8.3% true positive rate) appear to represent the inevitable statistical spillover from the negative control distribution; in this context, a high false-positive rate for synonymous guides serves as a diagnostic of near zero signal.

Collectively, our results suggest that single synonymous mutations in the human genome are 37.9-fold (95% CI: 22.16–81.48) less likely than missense mutations to impact fitness. Our findings of the rare fitness effects of synonymous mutations are consistent with and complementary to those of a recently published prime editing–based screen that tested ∼300 000 sites [18]. The high performance of positive controls in our study that induce strong fitness effects by eliminating start codons, creating stop codons, and introducing missense mutations supports the quality of our base-editing measurements and highlights the rarity of detectable synonymous fitness effects [11, 12].

Our findings contrast with observations from non-human model systems, particularly yeast [10], where synonymous mutations have been reported to produce fitness effects comparable to those of non-synonymous mutations. These differences might reflect fundamental distinctions in RNA biology between species. In yeast, strong codon bias and codon-optimality–dependent decay make mRNA stability and translation efficiency highly sensitive to synonymous changes [42, 43], and transcripts have short half-lives [44], amplifying codon-related effects on expression [44]. By contrast, human cells employ multiple overlapping decay pathways, and exhibit much longer mRNA half-lives, which buffer against single-site perturbations. Human transcripts also contain longer and more complex untranslated regions enriched for regulatory elements. Together, these layers of regulation could reduce the probability that a single synonymous substitution produces a measurable fitness effect in human cells. However, an alternative explanation is that the prior criticism of the yeast study [17] is valid.

While interspecies differences in RNA regulation may account for some of the discrepancy with prior studies, additional factors may also influence the likelihood of detecting synonymous effects in human systems. Our study has focused on measuring differences in cellular fitness conferred by sparse mutation(s) made by single sgRNAs, rather than the influence of epistatic interactions between mutations. When large swathes of codons within a gene are simultaneously and synonymously mutated, highly significant phenotypic changes have been observed [45, 46]. Moreover, our study did not probe changes in protein co-translational folding [47, 48], mRNA expression [49], stability and degradation [50, 51], miRNA-binding [16, 52], or other changes [53] associated with codon optimization, which can modulate molecular phenotypes without necessarily manifesting as measurable effects on cellular fitness. Finally, databases such as SynMall [54] catalog synonymous variants with predicted effects on gene expression, mRNA structure, and protein synthesis based on computational and literature evidence. Our results do not directly contradict these predictions, and the possibility for phenotypes to be affected by changes in gene expression, mRNA structure, and protein synthesis could be considered to explain the rare phenotypes that we observe.

We acknowledge several potential limitations. First, our study focuses on strongly essential genes as defined by DepMap in K562 and Jurkat cells. This choice reflects the logic of our experimental design: in a proliferation-based assay, only genes whose perturbation can produce fitness defects are informative. Essential genes are those most likely to reveal such effects; including non-essential genes in our library would introduce mutations to loci where no fitness consequence could be observed, regardless of mutation type, thereby diluting signal and adding experimental challenges without adding interpretive value. Consequently, if synonymous mutations were to produce widespread fitness effects in the human genome, cell level essential genes represent the context in which such effects would be most readily detected in cell culture. Our design ensured gene-level comparability by including missense controls in every essential gene tested. Direct codon-level pairing of synonymous and missense edits might be ideal, but it is not feasible because we require a 15-bp insulated editing window to eliminate potential bystander mutations at missense sites that could confound the interpretation of guides identified as “synonymous.” Of course, it should come as no surprise to the reader, that our assay does not capture phenotypes that may manifest during development, in specific tissues, or under non-standard growth conditions, though we think that there is no a priori reason to expect synonymous fitness effects to be more prevalent in these contexts than in the highly essential cellular growth gene set examined here. Finally, our likelihood model does not explicitly incorporate covariates such as gene expression level, local codon usage, or sgRNA-specific editing efficiency. In principle, these factors might influence the estimated fold-difference between mutation classes. We note that synonymous and missense sgRNAs in our study do not differ systematically in these measures, but we cannot exclude the possibility that unmeasured or unknown covariates contribute to the observed difference.

Taken together, our work constitutes a large-scale experimental test of synonymous mutation effects on cellular proliferation in essential genes in human cells. By excluding mutations within ±2 nucleotides of splice junctions, our study specifically interrogates the fitness consequences of synonymous changes at the codon level, independent of splicing disruption [51, 52]. In light of recent contrary claims from other studies, our results provide strong experimental evidence that many of the foundational results and widespread analysis methodologies in genetics, genomics, evolutionary biology, and medicine that assume synonymous neutrality are likely reasonable. This interpretation is consistent with other large-scale experiments performed with prime editors [18], and are especially true when likely splice modifying positions with synonymous effects on individual codons are computationally removed from the background set.

## Supplementary Material

gkag733_Supplemental_Files

## Data Availability

The base editor library sequencing data are deposited under Bioproject PRJNA1236376. All custom code used for analysis is available on GitHub (https://github.com/ryy1221/synSg) and Zenodo (https://doi.org/10.5281/zenodo.21109071). Additional data used in the study are available in the Zenodo database under the DOI identifier (https://doi.org/10.5281/zenodo.15241793).
