# Correction to ‘Synonymous mutations in essential genes infrequently produce fitness effects in human cell lines’

**DOI:** 10.1093/nar/gkag855

**Published:** 2026-09-02

This is a correction to: Yiyun Rao, Ivan Sokirniy, Edward O’Brien, Justin Pritchard, Synonymous mutations in essential genes infrequently produce fitness effects in human cell lines, *Nucleic Acids Research*, Volume 54, Issue 14, 12 August 2026, gkag733, https://doi.org/10.1093/nar/gkag733

In the **Materials and methods** section, the sentence “Before proceeding to correlation coefficient calculation, read counts were first normalized to log2reads per million using:” was incorrectly formatted as a heading. This has now been corrected.

The heading: “**Sgrna editing efficiency estimation**” has also been corrected to read: “**SgRNA editing efficiency estimation**”.

In addition, the resolution of Figure 2C was lower than intended in the published version. Figure 2C has been replaced with a higher-resolution image.

These corrections do not affect the results, discussion and conclusions presented in the article. The published article has been updated.

