# Supplementary material for: Synonymous mutations in essential genes infrequently produce fitness effects in human cell lines
Source: Nucleic Acids Res. 2026 Aug 5;54(14):gkag733. doi: 10.1093/nar/gkag733 (PMC13439131; doi:10.1093/nar/gkag733)
Supplement: Supplementary file 1 [file gkag733_supplemental_files.zip › SupplementaryInformation_revision (1) (1).docx]

Table of Contents

[Supplementary Methods	2](#_Toc195795329)

[Library Composition and Description	2](#_Toc195795330)

[PCR Conditions	3](#_Toc195795331)

[Supplementary Note	4](#_Toc195795332)

[Ghost Editor Screen	4](#_Toc195795333)

[Hit selection Criteria	5](#_Toc195795334)

[Identification of Potential Off-Target Edits	5](#_Toc195795335)

[Supplementary Figures	7](#_Toc195795336)

[References	20](#_Toc195795337)

# Supplementary Methods

Library Composition and Description **(**See Supplementary Table 3 and 4 for detailed sgRNA sequences**)**

***ABE Library:***

1. Synonymous sgRNA(n = 2134)
2. Missense sgRNA (n = 232)
3. Dual iSilence (n = 673): NG PAM iSilence library that can be targeted by both adenine and cytosine base editors across the whole genome. It is generated by tiling the start codon across common essential genes. This gave 673 total unique iSilence dual sgRNAs included in both ABE and CBE libraries.
4. Randomly selected synonymous and missense sgRNAs in neutral targeting genes (n = 196)
5. Randomly selected empty window sgRNAs in the essential targeting genes.(n = 407)
6. sgRNAs targeting AAVS1 and CCR5 & Non-targeting sgRNAs (n = 895)

***CBE library:***

1. Synonymous sgRNA (n = 3528)
2. Missense sgRNA (n = 367)
3. Dual iSilence Library as in ABE (n = 673)
4. iSTOP^1^ Library(n=902): sgRNAs disrupt the stop codon in essential genes across the whole genome
5. Stop sgRNAs in Target Essential Genes(n = 181)**:** Due to the cost of sgRNAs, we randomly selected 181 stop sgRNAs in the 128 targeted essential genes to ensure every gene had one corresponding stop sgRNA in the library.
6. Randomly selected synonymous and missense sgRNAs in neutral targeting genes (n = 231)
7. Randomly selected empty window sgRNAs in the essential targeting genes.(n = 458)
8. sgRNAs targeting AAVS1 and CCR5 & Non-targeting sgRNAs (n = 895)

## PCR Conditions

**Supplementary Method Table1: sgRNA library amplification PCR**

| Compoenents | For 50uL reaction |
| --- | --- |
| 10X Buffer for KOD hot Start | 5 |
| 25mM MgSO4 | 3 |
| dNTP | 5 |
| Fwd primer | 1.5 |
| Sense primer | 1.5 |
| Template | 8 |
| Polymerase | 1 |
| Water | 25 |

| Cycling |  |  |
| --- | --- | --- |
| 95 | 2min |  |
| 95 | 20s | 15 cycles |
| 55 | 10s |  |
| 70 | 10s |  |

**Supplementary Method Table2: sgRNA Ligation**

| Ligation component | Amount |
| --- | --- |
| 91bp sgRNA PCR insert | 4.3ng |
| Linearized lentiGuide-Puro vector | 150ng |
| Esp3I (10 U/μl) | 1uL |
| T7 DNA ligase (3000 U/ul) | 1uL |
| Esp3I reaction buffer (10x) | 2uL |
| ATP (10mM) | 2uL |
| ddH2O | to 20uL |

| Step | Temp |  |
| --- | --- | --- |
| 1 | 37 | 2hr |
| 2 | 20 | 20min |
| 3 | 37 | 5min |
| 4 | To step2, 9 cycles |  |
| 5 | 4C |  |

# Supplementary Note

## Ghost Editor Screen

To investigate the potential deaminase-independent effects in base editing, we first analyzed data from the Ghost Editor screen. Similar to the base editor screen, we defined hits as sgRNAs with adjusted p-values < 0.05 in both K562 and Jurkat cell lines. Strikingly, the Ghost Editor generated hits across all essential loci categories, including synonymous, missense, no-edit, and positive control (iSilence) sgRNAs, but not in non-essential loci. This was true in both our ABE and CBE libraries (Supplementary Figure 5A), indicating that Ghost Editor hits occur when the nickase is targeted to an essential locus, and underscoring the importance of performing the control. However, the magnitude of the hit rates in Ghost Editor screens remained consistently low and similar across sgRNA categories: 0.52% for synonymous (ABE) and 0.4% (CBE), 0.43% for missense (ABE) and 0.55% (CBE), and 0.25% for no-edit (ABE) and 0.66% (CBE). This low but consistent creation of hits suggests that some false positives in current editing screens are due to editing independent effects at essential loci, but that it is not a major source of false positives.

iSilence sgRNAs, which target the start codons of coding sequences, are positioned closest to the beginning of transcripts. Notably, the Ghost Editor exhibited the highest hit rates for iSilence sgRNAs (2.32% for ABE and 3.21% for CBE) among all sgRNA categories (Supplementary Figure 5B). Furthermore, a notable enrichment of shared hits between the Ghost Editor and base editor screens was observed for iSilence sgRNAs (Supplementary Figure 5C). In ABE, 86% (13/15) of Ghost Editor iSilence hits were shared with base editor hits, while in CBE, 40% (8/20) of Ghost Editor iSilence hits overlapped with base editor hits. By contrast, in other essential sgRNA categories, only synonymous sgRNAs in Ghost Editor screens demonstrated a 9.09% (1/11) overlap with ABE base editor hits, and no overlap was observed for missense sgRNAs These findings support the potential hypothesis that deaminase-independent transcriptional blocking effects may be a contributor to false-positive results in base editing screens.

Another intriguing observation was the increased hit rates for presumably neutral sgRNAs (synonymous and no-edits) in Ghost Editor screens compared to base editor screens, observed in both ABE and CBE libraries (Supplementary Figure 5B). This phenomenon may reflect looser binding dynamics of Cas9 in the absence of the deaminase domain. For example, TadA7.10, the deaminase in ABE8e, enhances positive charge density in its DNA-binding region, strengthening Cas9-DNA interactions^2^. Without the deaminase, Cas9 may exhibit weaker or less specific binding, leading to increased hits for neutral sgRNAs in Ghost Editor screens.

## Hit selection Criteria

(1) A candidate is considered significant if the adjusted p-value is < 0.05 in both cell lines between Day 0 and Day 12. (2) A For CBE, due to limited editing efficiency^3,4^, if there are no overlapping significant candidates between the two cell lines, we select candidates that are significant at both time points (D26 and D12) in K562. (3) The candidate must not be identified as a hit in the Ghost Editor screen (adjusted p-value < 0.05 in both Jurkat and K562).

## Identification of Potential Off-Target Edits

To assess potential off-target edits in the genome, we performed the same PSMB5 base editing experiment in two additional replicates. Cells expressing both the sgRNA and base editor were collected at two-time points: Day 5 and Day 17. These four samples, along with a control parental K562 CBEd strain collected before infection, were submitted to Novogene for whole-genome sequencing at 30X coverage.

Variant detection and annotation were performed by Novogene using GATK and ANNOVAR^5^, referencing both RefSeq and GENCODE databases to identify genomic regions affected by variants and their potential impact on protein function.

Due to the limitations of low-coverage sequencing, our analysis focused on single nucleotide polymorphisms (SNPs). Variants were prefiltered based on the following criteria:

1. The variant must be present in all four experimental samples (Day 5 and Day 17 of both replicates).
2. The variant must be of high quality (Phred score >60).
3. Both WT and mutated alleles must be present at the potential variant position.
4. The variant must not be detected in the parental K562 sample.
5. The variant must not be present in the DepMap WT K562 mutation dataset.

Following this prefiltering process, 25,424 candidate variants were identified. Among these, we prioritized 231 mutations in common essential genes. Among which 5 of those are exonic mutations (Supplementary Table 11), including five variants located within essential genes(VARS1 NM_006295 c.G2692A, CARS1 NM_001378140 c.1593T, HINFP NM_001351963 c.C860P, CTU2 NM_001318511 c.G1019A). To further refine the candidate list and identify mutations most likely to contribute to cell depletion, we applied a minor allele frequency (MAF) cutoff using the gnomAD^6^ database, retaining only variants with an observed gnomAD genome frequency of <0.01. This filtering resulted in two final candidate mutations located in VARS1 and CTU2, as well as our target PSMB5.

To validate these findings, we performed targeted sequencing on the identified VARS1 and CTU2 regions, along with the PSMB5 candidate region for the WGS samples. While edited alleles for VARS1 and CTU2 were confirmed, their allele frequencies remained unchanged between time points, indicating that these mutations did not contribute to significant cell depletion under the experimental conditions. At the same time, PSMB5 allele-specific edit consistently shows depletion. (Supplementary Table 10, Supplementary Figure 10).

**Supplementary Note Table1: WGS sequencing exonic off-target candidates (showing only top transcript)**

| CHROM | POS | REF | ALT | GeneName | ExonicFunc | AAChange | Allele Freq C2_S1 | Allele Freq C2_S2 | Allele Freq C2_E1 | Allele Freq C2_E2 | Priority | gnomad_genome_AF |
| --- | --- | --- | --- | --- | --- | --- | --- | --- | --- | --- | --- | --- |
| chr6 | 31780896 | C | T | VARS1 | missense SNV | VARS1:NM_006295:exon23:c.G2692A:p.E898K | 0.18461538 | 0.17857143 | 0.16326531 | 0.11627907 | H | . |
| chr11 | 3006910 | G | A | CARS1 | synonymous SNV | CARS1:NM_001378140:exon16:c.C1593T:p.P531P | 0.35483871 | 0.40540541 | 0.375 | 0.38461538 | L | 0.2595 |
| chr11 | 119134422 | C | T | HINFP | missense SNV | HINFP:NM_001351963:exon9:c.C860T:p.A287V | 0.26470588 | 0.34375 | 0.28571429 | 0.38709677 | L | 0.3588 |
| chr14 | 23033420 | G | A | PSMB5 | synonymous SNV | PSMB5:NM_001130725:exon2:c.C144T:p.G48G | 0.28571429 | 0.17647059 | 0.13043478 | 0.24137931 | L | . |
| chr16 | 88714665 | G | A | CTU2 | missense SNV | CTU2:NM_001318513:exon11:c.G1019A:p.R340Q | 0.4047619 | 0.18867925 | 0.425 | 0.2 | L | 0.092 |

# Supplementary Figures

Supplementary Figure 1

Workflow of the sgRNA screen. K562 and Jurkat cells were transduced with the sgRNA library on a separate vector (D0). Following the library introduction, replicates were again independently infected with ABE8e, CBEd, or a deaminase-null Ghost Editor. After infection and selection, cells were collected at the first endpoint (D12) for both ABE and CBE screens in K562 and Jurkat cells. The CBE screen duration in K562 was extended by an additional 14 days (D26) to allow for the potential to identify more CBE hits if necessary. Cells were then harvested for next-generation sequencing (NGS) to assess sgRNA representation at the baseline and Day 12 or Day 26 endpoint. Differential read counts were analyzed using DESeq2.

Supplementary Figure 2

(A) Replicate correlation coefficient for Jurkat and K562 sgRNA set in ABE between Day 0 and Day 12. Each dot is a sgRNA in the positive control set.

(B) Volcano plot for K562 and Jurkat positive control sgRNA sets, the x-axis is log2 fold change and the y-axis is the -log10 adjusted p-value. Each dot is a sgRNA in the positive control set. Red: p-value < 0.05.

Supplementary Figure 3

(A) Replicate correlation coefficient for Jurkat and K562 sgRNA set in CBE between Day 0 and Day 12. Each dot is a sgRNA in the positive control set.

(B) Volcano plot for K562 and Jurkat positive control sgRNA sets, the x-axis is log2 fold change and the y-axis is the -log10 adjusted p-value. Each dot is a sgRNA in the positive control set. Red: p-value < 0.05.

Supplementary Figure 4

(A) Replicate correlation coefficient for Jurkat and K562 sgRNA set in CBE between Day 0 and Day 26. Each dot is a sgRNA in the positive control set.

(B) Volcano plot for K562 and Jurkat positive control sgRNA sets, the x-axis is log2 fold change and the y-axis is the -log10 adjusted p-value. Each dot is a sgRNA in the positive control set. Red: p-value < 0.05.

Supplementary Figure 5

(A) Scatter plots showing sgRNA normalized log2 fold changes (x-axis: K562; y-axis: Jurkat) across various categories in the ABE and CBE libraries in the Ghost Editor Screen. Each data point represents a sgRNA. Categories include essential loci (synonymous, missense, no-edit, and iSilence) and non-essential loci (control sgRNAs and neutral sgRNAs).

(B) Bar plots representing square-root normalized hit rates (%) for the corresponding categories in panel A. Hit rates are shown separately for Ghost Editor hits (olive) and base editor hits (purple) in the ABE (left column) and CBE (right column) libraries.

(C) Venn diagrams illustrating the overlap of hits between Ghost Editor (olive) and base editor (purple) screens for the ABE (left column) and CBE (right column) libraries. Each row corresponds to the categories shown in panel A, highlighting shared and unique hits between Ghost Editor and base editor screens.

Supplementary Figure 6

­­­

(A) Single sgRNA validation of missense mutations. The percentage of GFP-positive cells over time is shown for K562 cells infected with sgRNA candidates (blue circles), matching no-edit sgRNAs (blue squares), an iSilence control sgRNA (blue downward triangles), or Ghost Editor sgRNAs (blue upward triangles) in cells expressing base editors (ABE8e or CBEd). Each panel represents a specific missense sgRNA tested in either the ABE or CBE library.

(B) Single sgRNA validation of synonymous mutations. The percentage of GFP-positive cells over time is shown, with annotations identical to panel A. Each panel represents a specific synonymous sgRNA tested in either the ABE or CBE library.

Supplementary Figure 7

The percentage of sequencing reads supporting each detected allele-specific edit at Day 5 and Day 17 for synonymous sgRNAs. Individual alleles are plotted with their corresponding read percentages to visualize their percentage over time. The values represent the observed allele frequency of edited mutations in a mixture of cells and don’t reflect editing efficiency. Allele-specific reads that > 1% of the total reads, and present in both Day 5 and Day 17 are analyzed and shown. The edited position is highlighted in yellow.

Supplementary Figure 8

The percentage of sequencing reads supporting each detected allele-specific edit at Day 5 and Day 17 for ABE missense sgRNAs. Individual alleles are plotted with their corresponding read percentages to visualize their percentage over time. The values represent the observed allele frequency of edited mutations in a mixture of cells and don’t reflect editing efficiency. Allele-specific reads that > 1% of the total reads, and present in both Day 5 and Day 17 are analyzed and shown. The edited position is highlighted in yellow.

Supplementary Figure 9

The percentage of sequencing reads supporting each detected allele-specific edit at Day 5 and Day 17 for CBE missense sgRNAs. Individual alleles are plotted with their corresponding read percentages to visualize their percentage over time. The values represent the observed allele frequency of edited mutations in a mixture of cells and don’t reflect editing efficiency. Allele-specific reads that > 1% of the total reads, and present in both Day 5 and Day 17 are analyzed and shown. The edited position is highlighted in yellow.

Supplementary Figure 10

The percentage of sequencing reads supporting each detected allele-specific edit at Day 5 and Day 17 for the potential off-target loci in CTU2 and VARS1, as well as the synonymous candidate PSMB5. Individual alleles are plotted with their corresponding read percentages to visualize their percentage over time. The values represent the observed allele frequency of edited mutations in a mixture of cells and don’t reflect editing efficiency. Allele-specific reads that > 1% of the total reads, and present in both Day 5 and Day 17 are analyzed and shown. The edited position is highlighted in yellow.

Supplementary Figure 11

A) Single sgRNA validation of additional synonymous sgRNA hits in ABE and CBE that have adjusted p-value < 0.2. The percentage of GFP-positive cells over time is shown for K562 cells infected with sgRNA candidates ,matching no-edit sgRNAs (grey), an iSilence control sgRNA (blue). The validated synonymous sgRNA hit in DDX54 was colored in red.

B) The percentage of sequencing reads supporting each detected allele-specific edit at Day 5 and Day 11 for the ABE candidate in DDX54, Individual alleles are plotted with their corresponding read percentages to visualize their percentage over time. The values represent the observed allele frequency of edited mutations in a mixture of cells and don’t reflect editing efficiency. Allele-specific reads that > 1% of the total reads, and present in both Day 5 and Day 17 are analyzed and shown. The edited position is highlighted in yellow.

Supplementary Figure 12

BE-Hive predicted sgRNA read fraction for ABE and CBE library. The predicted read fraction is on a scale of 0 to 1. 1 being 100% predicted. Synonymous sgRNAs (purple box) and Missense sgRNA (blue box) are shown. Rank-based Mann-Whitney U test was performed to determine if the missense and synonymous distribution are significantly different or not. P-values are shown.

Supplementary Figure 13

**ROC curve assessing screen performance across combined ABE and CBE libraries.** Receiver operating characteristic (ROC) curve showing the trade-off between true positive rate (TPR) and false positive rate (FPR) across significance thresholds (adjusted p-value cutoffs) for ABE and CBE iSilence positive controls and empty window negative control. The TPR was defined as the proportion of iSilence guides identified as significant in both K562 and Jurkat cell lines, while FPR was computed from empty window guides.

# References

1.	Billon, P. *et al.* CRISPR-mediated base editing enables efficient disruption of eukaryotic genes through the induction of STOP codons. *Mol Cell* **67**, 1068–1079 (2017).

2.	Zhu, H. *et al.* Directed-evolution mutations enhance DNA-binding affinity and protein stability of the adenine base editor ABE8e. *Cellular and Molecular Life Sciences* **81**, 257 (2024).

3.	Zuo, E. *et al.* Cytosine base editor generates substantial off-target single-nucleotide variants in mouse embryos. *Science (1979)* **364**, 289–292 (2019).

4.	Jin, S. *et al.* Cytosine, but not adenine, base editors induce genome-wide off-target mutations in rice. *Science (1979)* **364**, 292–295 (2019).

5.	Wang, K., Li, M. & Hakonarson, H. ANNOVAR: functional annotation of genetic variants from high-throughput sequencing data. *Nucleic Acids Res* **38**, e164–e164 (2010).

6.	Chen, S. *et al.* A genomic mutational constraint map using variation in 76,156 human genomes. *Nature* **625**, 92–100 (2024).
